# Influences of vertical differences in population emigration on mountainous vegetation greenness: A case study in the Taihang Mountains

**DOI:** 10.1038/s41598-018-35108-w

**Published:** 2018-11-16

**Authors:** Wei Li, Minghong Tan

**Affiliations:** 1 Key Laboratory of Land Surface Pattern and Simulation, Institute of Geographical Sciences and Natural Resources Research, Chinese Academy of Sciences, Beijing, 100101 China; 2 College of Resources and Environment, University of Chinese Academy of Sciences, Beijing, 100190 China; 3 International College, University of Chinese Academy of Sciences, Beijing, 100190 China

## Abstract

With the rapid advance of urbanization, rural population emigration has become a key factor that affects the man-land relationship in China’s mountainous areas and may have a huge impact on ecological restoration. This study used the NDVI in the growing seasons to analyze the variation trend of vegetation greenness at different elevations in the Taihang Mountains during 2000–2010, employing trend analysis method. Then, we selected 990 samples, each of which was a circular area with a radius of 3 km. On this basis, we quantitatively analyzed the contribution degree of population emigration to this variation trend after eliminating the influences of precipitation, temperature, and other factors. The results showed that rural population emigration was significant in the Taihang Mountains in the past 10 years, with a rural population emigration rate of up to 16.3%; The vegetation in the Taihang Mountains presented a trend of overall improvement, but local deterioration; The results of the regression analysis showed that population emigration had significantly impacts on vegetation greenness at 1% significance level and 1% of population emigration can increase the NDVI variation trend by 0.06%. Furthermore, the impact gradually weakened with increasing elevation.

## Introduction

The world is currently experiencing rapid urbanization, with a significant population transfer from rural areas to cities, especially in developing countries. China is the developing country with the largest population in the world and is currently experiencing the greatest large-scale urban and rural population migration in human history^1^. According to the fifth and sixth census data, China’s urbanization rate increased from 36.2% in 2000 to 50.0% in 2010, while the rural population declined from 784 million in 2000 to 663 million in 2010^2,3^.

The decrease of the rural population can reduce the pressure on ecological systems to a certain extent and plays a significant role in promoting the improvement of the vegetation conditions^1,4^. Empirical researches from central Mexico^5,6^, southern Brazil^7^, Puerto Rico^8^, El Salvador^9^, and Costa Rica^10^ have demonstrated that the reduction of population pressure played an active role in vegetation restoration. On the one hand, workforce emigration will result in an increase of farmland abandonment in the out-migrating areas^1,11^, thereby promoting natural vegetation conditions^12–14^. On the other hand, the reduction of population pressure alleviates the disturbance of human activities on vegetation, e.g., decreasing grazing intensities and reducing deforestation, which can promote the recovery of vegetation conditions^15^. In China, a number of studies have also found that the reduction of population pressure has positive effect on the ecosystem^1,16,17^.

The normalized difference vegetation index (NDVI) is an important indicator that reflects the vegetation conditions and one of the main parameters used for monitoring vegetation. This index has been used by scholars around the world to analyze the changes in vegetation conditions on different scales^18–22^. Some studies have shown that the global vegetation improved since the 1990s to the turn of this century^23^; this trend was particularly obvious in the middle- and higher-latitude regions in the northern hemisphere^24,25^. In China, the vegetation variation shows strong spatial heterogeneity^26–29^. Moreover, the reasons for vegetation variation have also attracted widespread attention. Studies in this area have selectively analyzed the influences of natural factors on vegetation greenness variations, for example, exploring the correlativity between the NDVI variation and the temperature or precipitation^22,30–32^. In recent years, some scholars have begun to explore the influences of human activities on vegetation variation^33–35^. For example, Cai *et al*.^16^ have analyzed the influence of population emigration on vegetation greenness variations using Pearson’s correlation analysis, but this single-factor correlation analysis cannot rule out the effects of other factors such as temperature and precipitation. To comprehensively consider the effects of various factors on the vegetation, Wang *et al*.^36^ have deducted the impacts of temperature and precipitation on the vegetation and used the residual error of regression equations to express the impact of human activities when they analyzed the influences of climate changes and human activities on vegetation greenness variations in the mountainous areas of southern China. However, this method can only indicate the positive and negative effects of human activities, but fails to distinguish the types, the intensity, and the contribution degree of human activities. In addition, Cao *et al*.^17^ evaluated the relative contributions of human activities, climate change, and social economic development to ecological restoration since the 1980s by using province-level social economy data and remote sensing data. Similarly, Lu *et al*.^26^ investigated vegetation variation and its driving force in China at provincial level. These researches collected data at provincial level, without considering the significant differences in the regional geographical environment within the provincial administrative region. Therefore, such studies will help us to understand the variation of vegetation conditions and the corresponding influencing factors, but they do not fully reflect the influences of the changes of artificial factors on the vegetation greenness on the premise of controlling natural background conditions at the grid level.

The mountainous area is one of the main landscape types in China, accounting for up to two thirds of the national area. Mountainous areas are often important water sources for plain areas, and the ecological environment is usually fragile, with serious water and soil losses. Therefore, the ecological environment problems in mountainous areas have become one of the foci in research fields such as geography and ecology. In recent years, population emigration in mountainous areas has been significant. This poses the question whether population emigration in mountainous areas can alleviate the ecological pressure of mountains. If so, to what extent and are there any differences in terms of different elevations?

The Taihang Mountains are located on the eastern edge of China’s secondary terrain ladder and represent a natural barrier and important water source for the North China Plain. The region has a high population density with frequent and significant human activities; the vegetation in this area is therefore subjected to human disturbance. Hence, this region also experiences serious soil and water losses; the rural residents are rather poor and the conflict of man-land relationship is sharp^37,38^. In recent years, there has been a massive population migration in the Taihang Mountains, which prominently manifests as a decrease of the rural permanent resident population. Therefore, it is of great importance to quantitatively investigate the influences of population pressure changes on vegetation greenness in the Taihang Mountains.

This article, taking the Taihang Mountains as an example, uses MODIS data to analyze the spatial and temporal variation characteristics of NDVI in this region. Considering the accuracy and comprehensiveness of the census data, the selected research period was 2000–2010. We selected a variety of variables, including natural and anthropogenic factors, to analyze the factors influencing vegetation change. Then, we carried out rasterization on all variables. On the basis of controlling the influences of natural environmental elements, we quantitatively analyzed the influences of population pressure change and land-use intensity change on the local vegetation greenness and the differences of these influences at different elevations and identified the contributions of human activities. This can not only help to understand the dynamic response mechanism of vegetation variations, but also provides a valuable reference for the governance and recovery of regional ecological environments, the formulation of land use policies, and a reasonable guide to population migration^1^.

## Results

### Spatio-temporal variation analysis of NDVI

**Figure 1:**
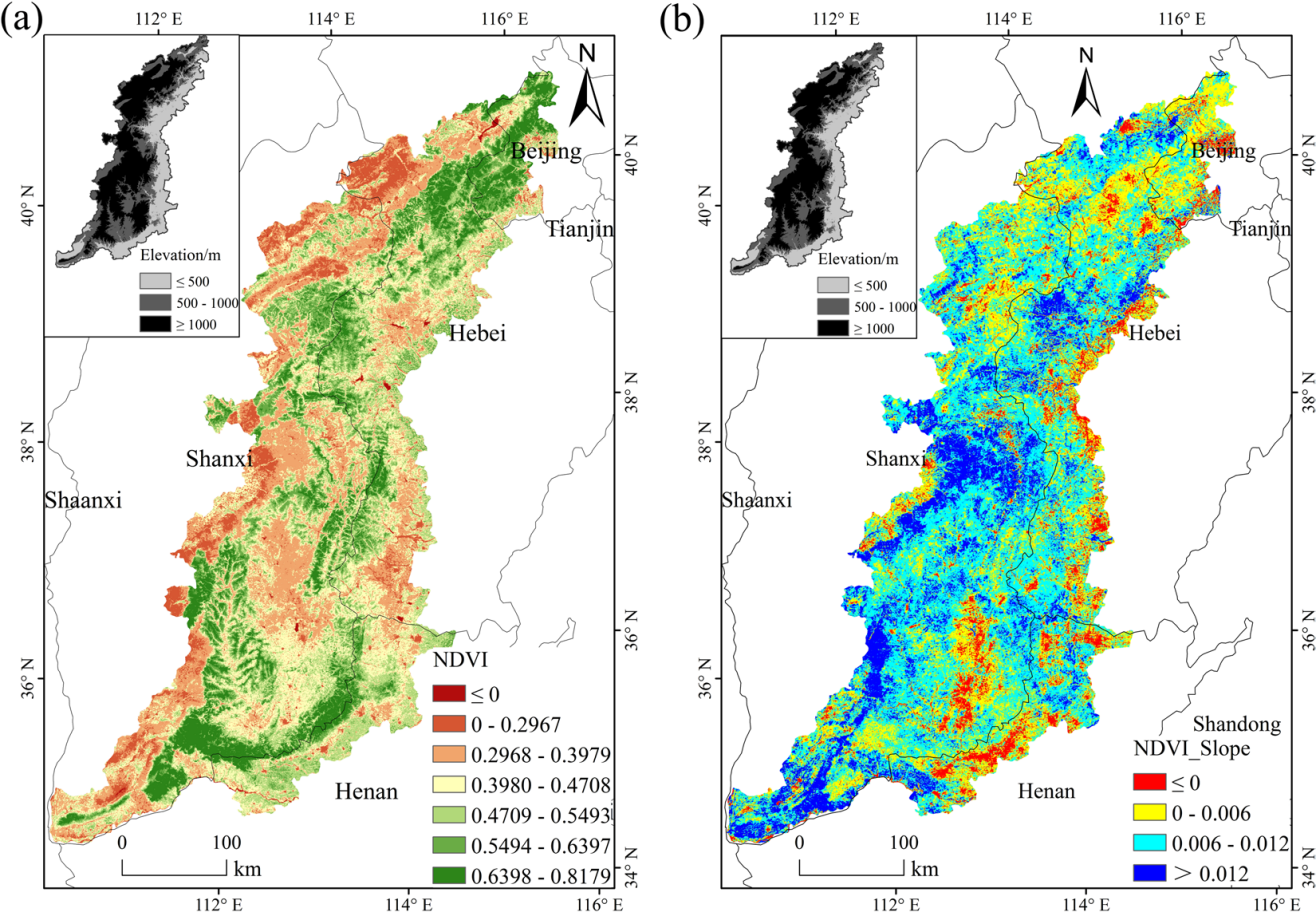
(**a**) Spatial distribution of the average NDVI values in the growing seasons in the Taihang Mountains in 2000; (**b**) Spatial distribution of the trend of NDVI variation in the Taihang Mountains from 2000 to 2010.

Figure 1(a) shows the spatial distribution of the average values of NDVI in the growing seasons in the Taihang Mountains in 2000. We observed large differences for the NDVI values at different elevations. Hereinto, NDVI values were higher at higher elevations, especially in the southern regions, while at lower- and middle-elevation regions, the NDVI values were lower.

Figure 1(b) shows the spatial distribution of the trend of NDVI variation in the Taihang Mountains from 2000 to 2010. The NDVI values in about 92.3% of the Taihang Mountains showed an increasing trend, in which the area of the regions with Slope values between 0 and 0.006 accounted for about 30.6% and that of the regions with Slope values between 0 and 0.012 accounted for about 76.7%. The regions with negative Slope values were mainly concentrated in the eastern lower-elevation regions adjacent to the North China Plain as well as some middle-elevation regions in the south. The NDVI values of these regions showed a declining tendency.

**Figure 2:**
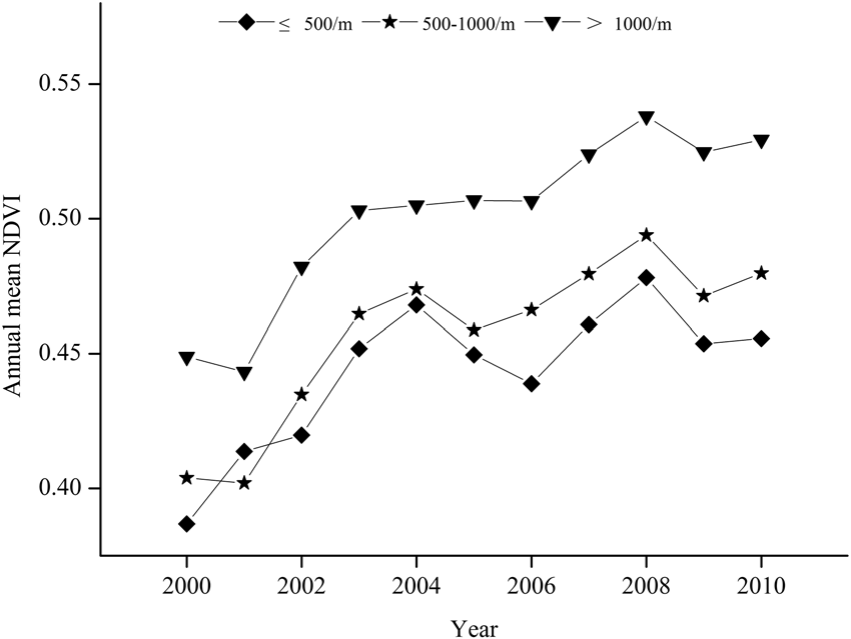
Average NDVI values at each elevation level of the Taihang Mountains from 2000 to 2010.

Overall, the average NDVI values in the growing seasons during 2000–2010 showed an increasing trend (Fig. 2). The trend varied across elevation. In lower-, middle-, and higher-elevation regions, the annual variation trend of NDVI values were 0.0062, 0.0076, and 0.0080, respectively, and the area with increased NDVI accounted for 86.1, 94.3, and 97.9% of the total area of lower-, middle-, and higher-elevation regions, respectively (Fig. 1b). The results indicate that the vegetation conditions in the Taihang Mountains have, overall, improved.

From the perspective of the annual variation trend, NDVI showed a significantly increasing trend during 2000–2004 at all regions with different elevations and, subsequently, a decreasing trend in the lower- and middle-elevation regions, but a stable level in the higher-elevation regions during 2004–2006. After 2006, NDVI values at all regions with different elevations first increased and then decreased; subsequently, they slowly recovered from the minimum value (Fig. 2).

### Influences of population emigration on NDVI variation trends

Prior to the model estimation, the collinearity of explanatory variables was tested by the Variance Inflation Factor (VIF). The results showed that the VIF values of all explanatory variables were below 5, with an average value of 1.85, indicating no any significant collinearity problem between explanatory variables (Table 1).

**Table 1:** Collinearity diagnosis in the model of overall areas of the Taihang Mountains.

Variables	VIF	Tolerance
Population density change	1.25	0.799
Land use intensity change	1.24	0.806
Slope temperature	1.18	0.845
Slope precipitation	2.90	0.345
Average gradient	1.57	0.637
Average aspect	1.12	0.896
Average elevation^*^	3.71	0.269
Mean VIF	1.85	

Note: * is the natural logarithmic of original average elevation.

For this study, we selected the NDVI variation trend from 2000 to 2010 as the explained variable, population emigration (population density change) as core explanatory variable, and land use intensity change, temperature variation trend, precipitation variation trend, gradient, aspect, and elevation as control variables; we analyzed the factors that affected the variation of vegetation greenness using the multivariate linear regression model. In the process of model specification, we successively introduced the explanatory variables to build a total of five models; the F values of each model are shown in Table 2. These five models all passed the F test.

**Table 2:** Models of impact of population migration on trends of NDVI variation in the Taihang Mountains.

Explained variable: Slope NDVI		Model 1	Model 2	Model 3	Model 4	Model 5	Standardized coefficients of Model 5
Explanatory variable	Population density change	−0.072***	−0.068***	−0.065***	−0.064***	−0.060***	−0.286
		(−12.87)	(−9.73)	(−8.88)	(−8.69)	(−7.74)	
		[−0.0722]	[−0.0685]	[−0.0655]	[−0.0637]	[−0.0597]	
	Land use intensity change		0.666	0.397	0.233	0.053	0.003
Control variable			(1.02)	(0.60)	(0.35)	(0.08)	
	Slope temperature^@^			0.299***	0.304***	0.158**	0.070
				(4.39)	(4.49)	(2.18)	
	Slope precipitation			0.068***	0.027	−0.095***	−0.155
				(3.60)	(1.29)	(−3.11)	
	Average aspect				0.201***	0.154***	0.105
					(4.46)	(3.38)	
	Average gradient				0.368**	0.005	0.001
					(2.11)	(0.02)	
	Average elevation^@@^					12.68***	0.311
						(5.55)	
	Constant	77.305***	72.575***	64.535***	28.849***	−31.428**	
		(68.85)	(68.73)	(35.25)	(3.67)	(−2.45)	
	Number of observations	990	990	990	990	990	
	Adjusted R−squared	0.120	0.122	0.152	0.172	0.198	
	AIC	9724.023	9724.247	9693.021	9673.441	9643.8	
	F	165.65	81.50	42.32	32.97	29.60	

Note: (1) The figures in () are t values; (2) The numbers in [] represent the marginal effect of population density change; (3) *, **, *** are coefficients different from zero at 10%, 5%, and 1% significance levels, respectively; (4) ^@^ is 1,000 times that of Slope Temperature, ^@@^ is the natural logarithmic of the original average elevation. (5) Standard error was adjusted for clusters in each sample; (6) All models were implemented by STATA13.0.

According to the related theories, population emigration positively impacts vegetation greenness. Table 2 shows the results of the influences of population emigration on vegetation greenness. Based on these results of the five models, population emigration can all significantly promote the increasing trend of NDVI in the entire Taihang Mountains area. Model 1 only introduced the population density change as the explanatory variable. At the 1% significance level, the population density change was significantly negative and the marginal effect was −0.072, namely 1% of population emigration can increase the NDVI variation trend by 0.072%. On the basis of Model 1, Model 2 introduced the land use intensity, and the population density change was still significantly negative at the 1% level, with a marginal effect of about −0.0685. On the basis of Model 2, we continued to introduce natural factors, including climate and topographic factors, and the marginal effects of population density change on NDVI variation trend was reduced. Nevertheless, population density change still had significantly negative impacts on the NDVI variation trend at a 1% significance level. 1% of population emigration can therefore increase the NDVI variation trend by 0.0597% in the model 5.

The standard partial regression coefficient reflects the degree of the direct effects of independent variables on the dependent variables under the condition of controlling other variables. In Table 2, the standard partial regression coefficients of variables in Model 5 demonstrate that the main influencing factors for the NDVI variation trend in the Taihang Mountains in the last 10 years include elevation, population density change, variation trend of precipitation, average aspect and variation trend of temperature (these factors are arranged according to their influencing degrees from high to low). From the perspective of influencing direction, the population density change had a significantly negative influence on the NDVI variation trend, whereas the average elevation, average aspect, and temperature change trend had a significantly positive influence on the NDVI variation trend.

In Table 2, the standard partial regression coefficients demonstrate that elevation had the largest contribution rate to the NDVI variation trend. To this end, we built three models for the higher-, middle-, and lower-elevation regions separately to further explain the trend of vegetation variations. From the perspective of all elevation scopes, there was a significant difference in the influence of population emigration on the NDVI variation trend. It is clear that, in the past decade, population emigration had significant impact on vegetation greenness in the lower- and middle-elevation regions, while there was no significant impact of population emigration on vegetation greenness in higher-elevation region (Table 3).

**Table 3:** Explanatory model for trend of NDVI variation in regions with lower, middle, and higher elevation.

Explained variable: Slope NDVI		Model 6 Lower elevation	Model 7 Middle elevation	Model 8 Higher elevation
Explanatory variable	Population density change	−0.055***	−0.073***	−0.048
		(−6.83)	(−6.03)	(−1.47)
		[−0.0545]	[-0.0734]	[−0.0480]
Control variable	Land use intensity change	0.155	−0.689	2.15***
		(0.25)	(−0.33)	(3.04)
	Slope temperature^@^	0.093	0.178	0.468***
		(0.70)	(1.52)	(4.35)
	Slope precipitation	1.02***	−0.054	-0.089
		(4.21)	(−0.59)	(−2.66)
	Average aspect	0.090	0.133	0.173**
		(1.01)	(1.58)	(2.57)
	Average gradient	0.497	0.812***	−1.53***
		(0.90)	(2.69)	(−5.47)
	Constant	31.056**	40.313 **	76.501 ***
		(2.19)	(2.52)	(5.94)
	Number of observations	245	324	421
	Adjusted R−squared	0. 379	0.160	0.201
	F	25.65	16.22	14.78

Note: (1) The figures in () are t values; (2) The numbers in [] represent the marginal effects of PDC; (3) *, **, *** are coefficients different from zero at 10%, 5%, and 1% significance levels, respectively; (4) ^@^ is 1,000 times that of Slope Temperature. (5) Standard error was adjusted for clusters in each sample; (6) All models were implemented by STATA13.0.

In the lower-elevation regions, the main factors that significantly affected the NDVI variation trend in the Taihang Mountains in the recent 10 years include population density change and precipitation change (Table 4), according to their influencing degrees from high to low. In middle-elevation regions, population density change and average gradient can affect the NDVI variation trend significantly at the 1% significance level. From the perspective of the influencing direction of fit coefficients, the factors that significantly affect the trend of NDVI variation all have a positive relation with the NDVI variation trend, except the population density change. Thus, we know that in the past decade, population emigration in the lower- and middle-elevation regions had significantly negative influences on the vegetation conditions of the Taihang Mountains, whose marginal effects were −0.055 and −0.073, respectively.

**Table 4:** Standardized coefficients of explanatory models for trend of NDVI variation in regions with lower, middle, and higher elevation of the Taihang Mountains.

Variable	Lower elevation	Middle elevation	Higher elevation
Population density change	**−0**.**407**	**−0**.**348**	−0.071
Land use intensity change	0.015	−0.033	**0**.**111**
Slope temperature^*^	0.041	0.084	**0**.**193**
Slope precipitation	**0**.**336**	−0.032	−0.131
Average gradient	0.071	**0**.**152**	**−0**.**272**
Average aspect	0.053	0.094	**0**.**119**

Note: * is 1,000 times that of Slope Temperature.

In the higher-elevation regions, population emigration had no significant impact on NDVI, whereas land use intensity and natural background had relatively significant influences. Among them, the temperature change trend, gradient, and land use intensity affected the NDVI variation trend at the 1% significance level, while the aspect affected the NDVI variation trend at the 5% significance level. The influence degrees of these factors from high to low follow the sequence of gradient, temperature variation trend, aspect, and land use intensity change. Table 3 shows that in higher-elevation regions, land use intensity had significantly positive influences on the NDVI trend. This indicates that the increase of land use intensity can promote the improvement of vegetation conditions to some extent.

Overall, the factors affecting the variation trend of vegetation greenness in Taihang Mountains showed a clear trend: at lower elevations, vegetation greenness was mainly influenced by population emigration; with the rise of elevation, the influences of natural factors become more and more significant, while the factor of population emigration began to recede.

## Discussion

In the context of rapid urbanization in China, rural population emigration is significant, and population change has significant influences on the regional environment^39,40^. From 2000 to 2010, the rural population emigration phenomenon in the Taihang Mountains was significant, with a decrease of 16.3%. In this paper, based on the MODIS data, we selected the Taihang Mountains as study region and used the population density change as the indicator to reflect population migration. After all the affecting factors were rasterized, we carried out statistical analyses on the samples selected at higher, middle, and lower elevations, on the basis of considering the natural elements (including temperature, precipitation, gradient, and aspect), and quantitatively analyzed the influences of population migration factors on vegetation greenness (NDVI).

**Figure 3:**
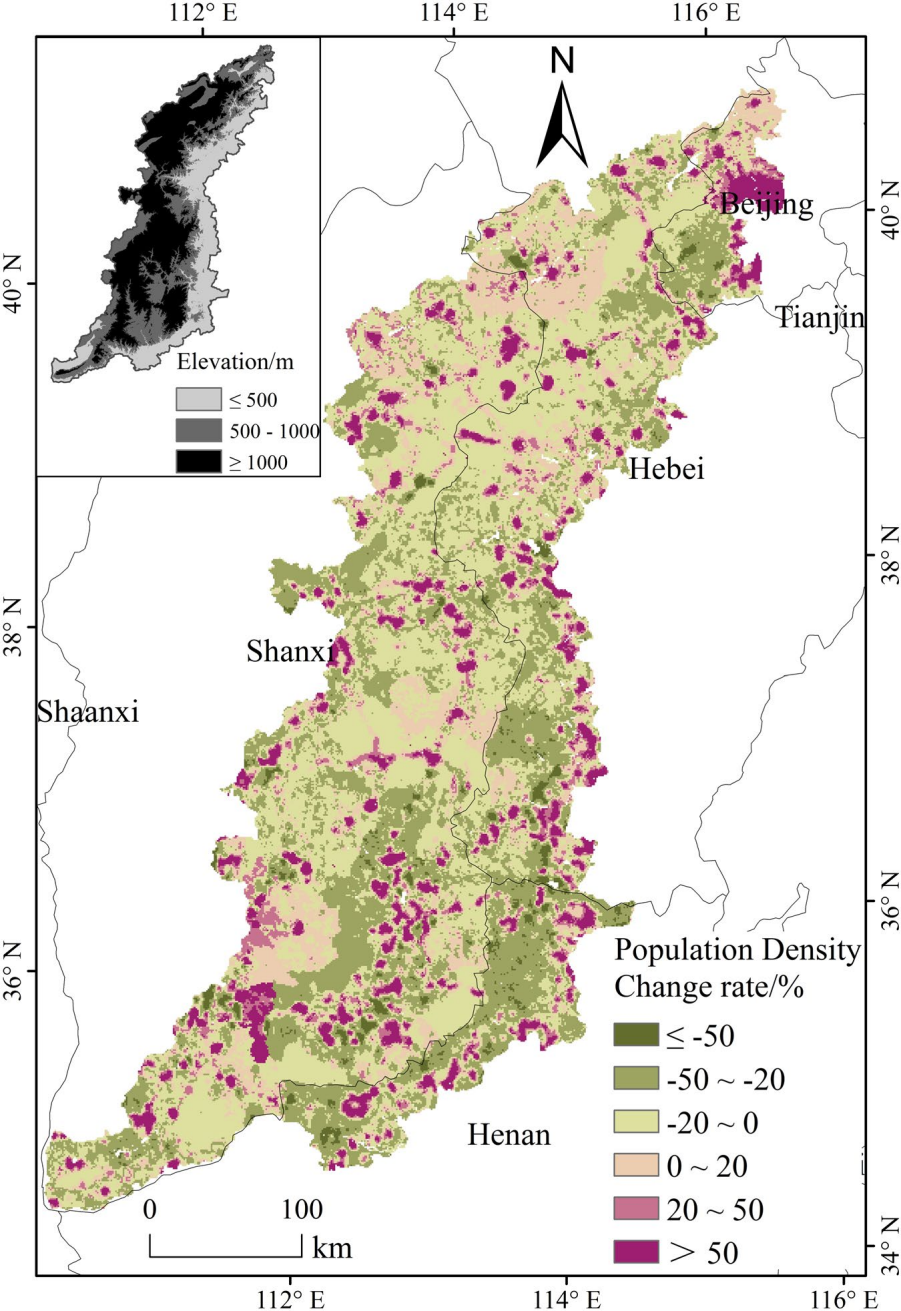
Spatial distribution of population density change rate during the period 2000–2010 in the Taihang Mountains.

The results showed that the influences of population emigration on vegetation greenness gradually weakened from lower to higher elevations. This trend was closely related with rural population emigration. Census data showed that the rural population decrement rates were 18.9, 10.6, and 8.7% in lower-, middle-, and higher-elevation regions, respectively (Fig. 3). From lower-elevation to higher-elevation regions, the amplitude of the population decline gradually decreased; in addition, the influencing degree of population emigration on the vegetation greenness also gradually decreased.

In higher-elevation regions, however, land use intensity had a remarkable positive influence on the dependent variables. Specifically, the increase of land use intensity can promote the increase of vegetation greenness. This kind of situation could be observed in higher-elevation regions of the Taihang Mountains, most likely due to the large area of barren hills in such regions. In recent years, for the purposes of water storage, flood control, and water and soil conservation, forestation and grass breeding have been promoted in the hillside fields^41^, which plays a positive role in increasing the vegetation greenness in this region. However, Models 6 and 7 show that the land use intensity in lower- and middle-elevation regions had no significant impact on the dependent variables. This may be related to the definition of land use intensity. In this paper, land use intensity was calculated using Eq. (2), which divides the land use status into four different utilization grades. The discontinuity of variables cannot fully reflect the influences of land use.

In addition, vegetation variations are also influenced by other human activities, such as land use policies, vegetation management strategies, etc.^42,43^. In particular since the 1990s, the massive ecological conservation and afforestation projects have induced more significant effects on vegetation restoration^44^. In this study, the variable of land use intensity reflects the influences of the “Grain for Green Policy” on land use change to a certain extent. However, this variable cannot fully reflect the influences of related policies on vegetation restoration, and it remains to be further improved in subsequent research.

Existing researches have rarely analyzed the improvement effects of the changes in human activities on vegetation conditions on the basis of controlling natural elements. In this article, we firstly rasterized all explanatory variables to obtain the raster data, including temperature, precipitation, gradient, elevation, population, and land use intensity. Then, on the basis of controlling other factors, we quantitatively analyzed the influences of population pressure and land use intensity change on the change trend of vegetation greenness by using the multivariate linear regression model. In addition, previous studies^16,17,26^ have mostly organized data based on provincial or county-level administrative units to analyze the influencing factors for the variations of vegetation greenness. For example, to investigate the influences of China’s social and economic factors on vegetation variation, Lu *et al*.^26^ have measured the influences of population pressure on vegetation brownification on the provincial scale by using the correlation analysis method. Similarly, Cai *et al*.^16^ have investigated the driving factors for forest restoration in the Karst regions of south China and analyzed the correlation relationship between population emigration and NDVI variation on the county scale. However, generally, the provincial administrative unit has a large area and contains a variety of geomorphic types, which means there are significant differences in the regional geographical environment within the province. Similarly, in the county-level administrative unit, it is difficult to reasonably distinguish the regional geographical environment with significant differences, especially in mountainous areas with significant terrain differences. In this study, the influencing factors were matched to the grid level, and the sample regions were selected randomly and equably on this basis. Such an approach helps to mimic the actual situation.

Li *et al*.^1^ have investigated the vegetation variation in rural and pastoral areas in Inner Mongolia and argued that artificial factors had important influences on vegetation coverage on the short-term scale. By contrast, in our study, the results showed that the influences of natural factors are greater than those of artificial factors in the Taihang Mountains, especially in remote higher elevation areas.

## Material and Methods

### Study area

**Figure 4:**
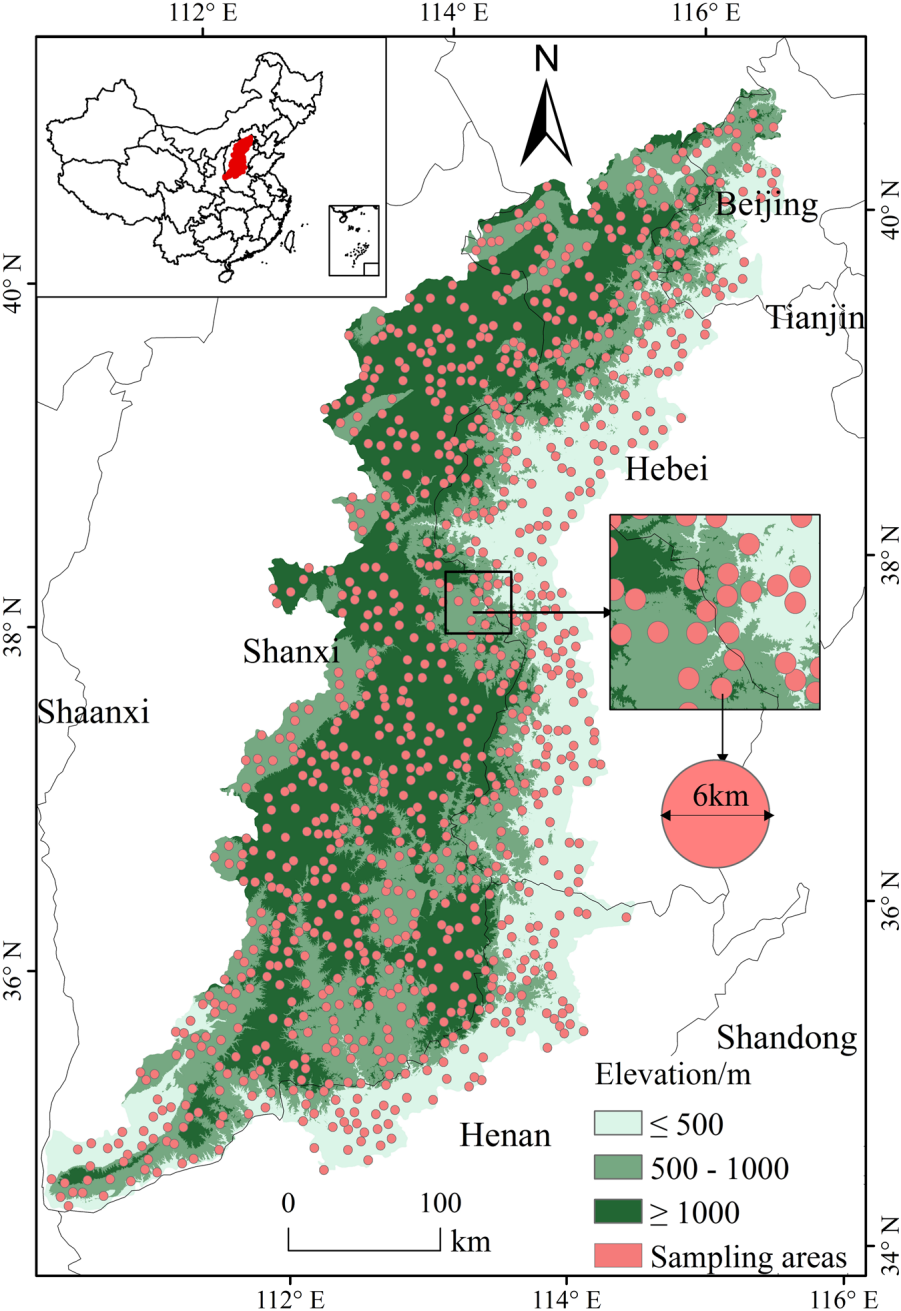
DEM map and spatial distribution of sample areas in the Taihang Mountains.

The Taihang Mountains cover an area of about 127,000 km^2^ and are located at the junction of four provincial administrations, i.e., Beijing, Hebei, Shanxi, and Henan (Fig. 4). The region contains 101 counties and represents an important geographic boundary for the Loess Plateau and the North China Plain. Average elevation is 1,000–2,000 m. The climate is warm temperate semi-humid continental monsoon climate, with four distinctive seasons. Winters are cold and dry, while summers are hot and rainy. Annual average temperature is around 10 °C, and annual rainfall is about 600 mm^45^. The natural vegetation includes aquatic vegetation, scrub-grassland, alpine meadow, broad-leaved forest, coniferous forest, etc.^46^. The vegetation has obvious seasonal characteristics and vertical zonal features.

### Data description

The data involved in our research mainly included NDVI data, DEM data, land use data, meteorological data, census data, and night-time light image data.

The spatial resolution of NDVI data was 250 m, with a time resolution of 16 d and a time span from 2000 to 2010. The NDVI data were obtained from the MODIS vegetation index product MOD13Q1 provided by the NASA platform^47^. The product was synthesized through image stitching, projection transformation, and mask machining on the basis of the data from Terra Star Three Level product MOD13Q after finishing the S-G filter. The monthly NDVI values were calculated by the maximum synthesis method. In this paper, we employed the NDVI monthly values from April to October in each year to calculate the average NDVI values in the growing seasons as the average value of the NDVI of the current year.

The DEM data are the SRTMDEM data in the territory of China, provided by International Scientific & Technical Data Mirror Site, Computer Network Information Center, Chinese Academy of Sciences^48^, with a spatial resolution of 90 m. The DEM data in the study area were masked from the DEM map of China. According to the elevation, the study area was divided into three sub-regions: lower-, middle-, and higher-elevation regions, namely the region lower than 500 m, the region between 500 and 1,000 m, and the region higher than 1,000 m (Fig. 4). The three sub-regions had a similar area.

The land use data in 2000 and 2010, with a 100 × 100 m spatial resolution, were obtained from the time series of land ecosystem classification dataset of china in Five-Year increments, published by the Global Change Research Data Publishing and Repository, Institute of Geographical Sciences and Natural Resources Research, Chinese Academy of Sciences^49^.

Meteorological data were obtained from the monthly value data set of China’s ground climatic standard values, provided by the China Meteorological Data Service Center^50^; the time span was 2000–2010. Similar to the calculation methods of annual average NDVI, we used the data in the growing seasons of each year to replace the climatic data of the current year.

Census data were obtained from the county-level statistics of the fifth and sixth demographic census by the National Bureau of Statistics^2,3^. Night-time light images were obtained from DMSP - OLS^51^. By simulating the relationship between the light intensity and population density, as well as some other factors (i.e., residential land proportion and cultivated land proportion), the population of permanent residents from the census data was matched on the county level^52^. Then the quantitative relationship between nighttime-light intensity and population density was extended to the grid scale.

### Trend analysis of NDVI and meteorological data

In this study, the trend analysis method was used to analyze the variation trends of NDVI and natural factors (annual average temperature and annual total precipitation) during this study period^53^. Namely, unary linear regression analysis was carried out on the NDVI, average temperature^54^, and precipitation values^55^, using time as independent variable. If the regression coefficient is negative, namely the slope is less than zero, the dependent variable shows a decreasing trend during the study period. On the contrary, if the slope is larger than zero, the corresponding variable shows an increasing trend^1,16,56,57^. The computation formula is as follows:

1 $$NDVI\_Slope=\frac{y\sum _{i=1}^{y}i\cdot NDV{I}_{i}-\sum _{i=1}^{y}i\sum _{i=1}^{y}NDV{I}_{i}}{y\sum _{i=1}^{y}{i}^{2}-{(\sum _{i=1}^{y}i)}^{2}}$$

where *NDVI_Slope* is the variation trend of NDVI during the study period; *y* is the number of years during the study period, and *y = *11 in this study; *i* represents the *i-th* year, ranging from 1 to *y*; $NDVI$ is the variable to be analyzed, including annual average NDVI, annual average temperature, and annual total precipitation.

### Interpolation of meteorological data

Surface monthly temperature and precipitation data in the 0.5° × 0.5° grid data set were obtained by spatial interpolation based on the temperature and precipitation data provided by 2,472 national meteorological stations in China, using the Thin Plate Spline (TPS) method in the specialized meteorological data interpolation software ANUSPLIN (The Australian National University, Canberra, ACT, Australia). In this paper, by using the TPS method of this software^58,59^, under the assistance of DEM data of 1 km, we carried out interpolation and obtained the monthly meteorological data set, with the resolution of 1 × 1 km.

### Calculation of land use intensity

According to the comprehensive analysis method proposed by Liu *et al*.^60–63^, the land use types can be divided into four levels, namely i) unused land (with a index of 1), including idle land and hard-to-use land, ii) forest-grass land (with a index of 2), including forestland, grassland, iii) agricultural land (with a index of 3), including cultivated land, garden land, and artificial grassland, and iv) urban settlement land (with a index of 4). The calculation formula for the land use intensity index is as follows:

2 $$\begin{array}{c}LUI=100\times \sum _{1}^{m}{G}_{i}\times {N}_{i}\\ LUI\in [100,400]\end{array}$$

where *LUI* represents the land use intensity comprehensive index, *m* is the number of levels of land use intensity, *G*_*i*_ refers to the grading index of the *i*-th level of land use intensity, and *N*_*i*_ refers to the percentage of the *i*-th level of land use area. Since this article mainly focuses on the influences of rural population emigration on the vegetation conditions in rural areas, urban settlement land, nature reserve areas, and water areas were not considered.

### Selection of influencing factors

In this paper, explanatory variables were selected from two aspects of natural factors and human activities^15^ (Table 5). In terms of natural factors, in order to reduce the influences of the large differences in temperature and precipitation interannual variation, we selected the change trends of the annual total precipitation and annual average temperature during the study period as analysis indicators. Meanwhile, gradient and aspect determine the site conditions for vegetation growth to a certain extent and were therefore also included in the explanatory variables. Land use intensity can quantitatively reveal the comprehensive level and change trend of regional land use. In the Taihang Mountains, the natural variation of population (birth and death rates) accounts for an extremely low proportion in the regional population variation. For instance, according to the census data, the natural variation rate of population in Anyang County of Henan province was 6.2% in 2000 and 6.5% in 2010, and the population variation in different years presents a development trend of low birth, low death, and low growth. In contrast, the decrease rate of the rural population in this county was 44.5% from 2000 to 2010. Consequently, population variation is represented by population emigration, while spatial population change is represented by population density change. Hence, in terms of human activity factors, we selected indicators from two aspects of population density and land use intensity.

**Table 5:** Main indicators impacting the changes of vegetation greenness in the Taihang Mountains.

	Factor	Description	Variable	Index	Unit
		Natural factors			
Control variable	Temperature	Variation in trend of temperature for 2000-2010, calculated using Equation (1)	Slope temperature	ST	—
	Precipitation	Variation in trend of precipitation for 2000–2010, calculated using Equation (1)	Slope precipitation	SP	—
	Gradient	Average gradient	Average gradient	AG	Degree
	Aspect	Average aspect	Average aspect	AA	—
	Elevation	Average elevation	Average elevation	AE	Meters
		Human activities			
	Land use	Land use intensity change from 2000 to 2010, calculated by the formula (2)	Land use intensity change	LUIC	—
Explanatory variable	Population	Population density change from 2000 to 2010	Population density change	PDC	Inhabitants/m²

### Selection of study samples

In this study, the samples were uniformly selected by the random sampling method (The sample numbers for higher, middle, and lower elevations were 245, 324, and 421, respectively). The scope of each sample was a circular area with a radius of 3 km (Fig. 4), without overlap between samples.

### Analysis of the multivariate linear regression model

The explanatory model for vegetation spatial variation in the Taihang Mountains was built by using the multivariate linear regression method. The dependent variable was the NDVI variation trend, with *n* independent variables, including natural factors and human activity factors, i.e., annual average temperature and annual total precipitation variation trends, average gradient and aspect, population density, and land use intensity change. The linear regression expression is given in Eq. (3):

3 $$NDVI\_slope={\alpha }_{1}PDC+{\alpha }_{2}LUIC+{\alpha }_{3}ST+{\alpha }_{4}SP+{\alpha }_{5}AG+{\alpha }_{6}AA+{\alpha }_{7}AE+\varepsilon ,$$

where ${\alpha }_{1},{\alpha }_{2,}{\alpha }_{3,}\cdot \cdot \cdot ,{\alpha }_{7}$ are the coefficients of the multivariate linear regression equation, *ε* is the standard error including the unobservable factors, and the meanings of *PDC*, *LUIC*, *ST*, *SP*, *AG*, *AA*, and *AE* are given as Table 5. Table 6 gives the statistical description for each variable of the 990 samples of the Taihang Mountains.

**Table 6:** Summary statistics of variables of the vegetation change explanatory model for the Taihang Mountains.

Variable	N	Minimum	Maximum	Mean	S.D
Population density change	990	−355.44	1022.94	10.23	126.88
Land use intensity change	990	−25.64	10.85	−0.46	1.91
Slope temperature*	990	−33.07	37.68	8.91	15.32
Slope precipitation	990	−8.45	271.41	76.55	54.26
Average gradient	990	0.48	24.36	10.42	6.61
Average aspect	990	84.55	286.12	173.77	23.82
Average elevation**	990	3.02	7.64	6.52	0.79
NDVI_Slope***	990	−38.45	1875.53	71.57	32.07

Note: (1) The total number of samples is 990; (2) * is 1,000 times that of Slope Temperature, ** is the natural logarithmic of original Average Elevation, and *** is 10,000 times that of NDVI Slope; (3) all operations were implemented by Stata 13.0.
